# Risk–Treatment Discordance and Fatal Cardiovascular Disease: A Nationwide Analysis Using PREVENT Thresholds

**DOI:** 10.1016/j.ajpc.2026.101711

**Published:** 2026-06-20

**Authors:** Adam W. Potter, Caroline E. Murphy, Bradley J. Petek, Christopher L. Chapman, Karl E. Friedl, Timothy W. Churchill, Nisha Charkoudian

**Affiliations:** a U.S. Army Research Institute of Environmental Medicine, Natick, Massachusetts, USA; b Sports Cardiology Program, Knight Cardiovascular Institute, Oregon Health & Science University, Portland, Oregon, USA; c Capital Cardiology Associates, Albany, New York, USA; d Cardiovascular Performance Program, Heart and Vascular Institute, Massachusetts General Hospital, Boston, Massachusetts, USA

**Keywords:** CVD, Cardiovascular disease, Mortality, Risk factors, Prevention

## Abstract

**Background:**

The 2026 multisociety dyslipidemia guideline adopted the PREVENT-ASCVD equations as the primary tool for identifying adults eligible for primary-prevention lipid-lowering therapy (LLT). The population-level treatment gap under this updated framework, and its relationship to fatal cardiovascular disease (CVD), have not been quantified.

**Methods and Results:**

Among 39,277 U.S. adults aged 30–79 years, 1,606 cardiovascular deaths occurred during follow-up; 38.5% occurred among individuals without baseline diagnosed CVD. Per-person risk was substantially higher among individuals with established CVD than among those without baseline CVD. Among 22,505 participants without baseline CVD with calculable PREVENT-ASCVD risk, 23.6% met the intermediate-or-higher risk threshold (≥5%), of whom 67.1% were not receiving LLT. At the borderline-or-higher threshold (≥3%), 35.9% met criteria, of whom 71.2% were untreated. Although per-person risk was substantially higher among individuals with established CVD (11.54 vs 0.97 per 1,000 person-years; *P* < 0.001), 38.5% (95% CI 35.2–41.8) of all cardiovascular deaths occurred in individuals without prior diagnosed CVD. Among individuals without baseline CVD, male sex, current smoking, and higher systolic blood pressure were independently associated with fatal CVD; diabetes was not independently associated after adjustment.

**Conclusions:**

A majority of U.S. adults meeting contemporary PREVENT-ASCVD thresholds for LLT consideration are not receiving therapy, and a substantial share of fatal CVD occurs in this undertreated, primary-prevention population. Retrospective application of contemporary PREVENT thresholds suggests that substantial numbers of U.S. adults who subsequently experienced fatal cardiovascular disease would have met current treatment eligibility criteria yet were not receiving lipid-lowering therapy.

**Figure:**
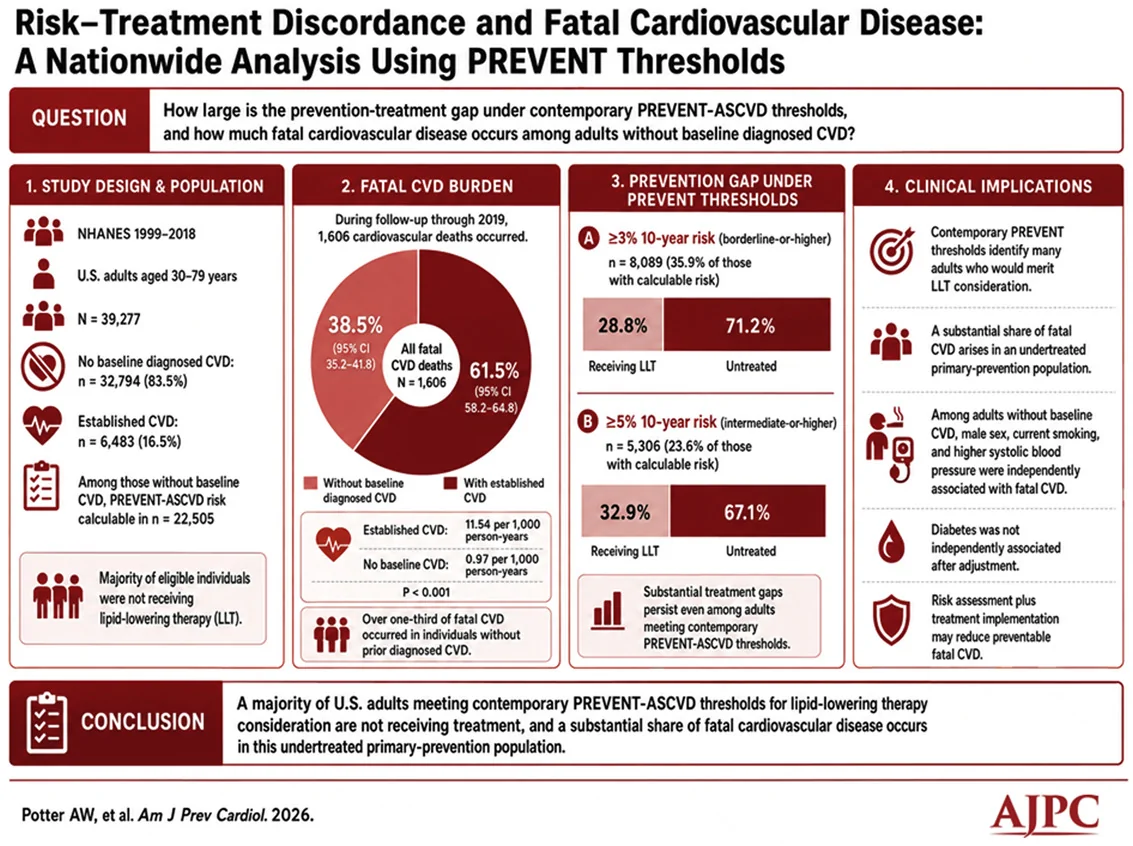
Central Illustration. Risk–Treatment Discordance and Fatal Cardiovascular Disease Legend: Among U.S. adults aged 30–79 years in NHANES 1999–2018 linked to mortality follow-up through 2019, 38.5% of fatal cardiovascular disease (CVD) events occurred among individuals without baseline diagnosed CVD. Among participants without baseline CVD and calculable PREVENT-ASCVD risk, 35.9% met borderline-or-higher (≥3%) risk thresholds and 23.6% met intermediate-or-higher (≥5%) risk thresholds for lipid-lowering therapy (LLT) consideration, yet most eligible individuals were untreated. Percentages represent survey-weighted estimates. Incidence rates are expressed per 1,000 person-years.

## Introduction

Cardiovascular disease (CVD) remains the leading cause of mortality globally, accounting for an estimated 20.5 million deaths annually [1]. In the United States alone, CVD was responsible for 931,578 deaths in 2021, approximately one death every 34 s [2]. Over recent decades, improved recognition of structural, metabolic, and electrophysiological risk factors has expanded preventive and therapeutic options, contributing to meaningful reductions in morbidity and mortality among individuals with diagnosed disease. However, fatal cardiovascular events frequently occur before clinical recognition of underlying disease, suggesting that substantial opportunities for earlier identification and preventive intervention remain.

A central challenge in cardiovascular prevention is that fatal cardiovascular events may occur before clinical recognition of disease, leaving limited opportunity for secondary prevention. Many individuals who experience fatal CVD have no prior diagnosis, reflecting a disconnect between underlying risk and clinical recognition. This pattern aligns with the prevention paradox, in which the majority of events arise from a large population with moderate or unrecognized risk rather than from a smaller group of individuals at high, clinically identified risk [[3], [4], [5]]. While individuals with established CVD have substantially higher absolute risk, the broader population without diagnosed disease contributes a large proportion of total cardiovascular mortality.

This study retrospectively applies the contemporary PREVENT-ASCVD framework to a historical U.S. cohort (NHANES 1999– 2018) to perform a counterfactual analysis of treatment gaps. The 2024 release of the American Heart Association PREVENT-ASCVD equations [6], and their formal adoption in the 2026 ACC/AHA multisociety dyslipidemia guideline [7], have replaced the Pooled Cohort Equations as the standard tool for primary-prevention risk stratification. PREVENT incorporates kidney function, metabolic risk, and social determinants and reclassifies a substantial portion of the population relative to prior risk calculators, raising new questions about how many individuals now meet contemporary thresholds for lipid-lowering therapy (LLT) and how many of those individuals are actually treated. To our knowledge, no nationally representative analysis has yet characterized the size of the LLT treatment gap under the PREVENT-aligned 2026 framework or examined how that gap relates to downstream fatal CVD.

Underlying this phenomenon is the well-established observation that cardiometabolic risk factors, including hypertension, dyslipidemia, smoking, and central adiposity, often progress without overt symptoms. These “silent” risk factors require proactive identification and management, yet gaps persist in their recognition and treatment. Although disparities in healthcare access, socioeconomic status, and other structural factors have been associated with differences in cardiovascular outcomes [1,2,8], it remains unclear to what extent fatal events occurring without prior diagnosis reflect barriers to access versus missed opportunities for risk detection and treatment within the healthcare system.

Here we provide one of the first nationally representative estimates of the LLT treatment gap under the PREVENT-aligned 2026 framework, and link this gap to fatal cardiovascular outcomes in the same population. We retrospectively applied contemporary PREVENT-based treatment thresholds to historical NHANES cohorts to evaluate the extent to which individuals who subsequently experienced fatal cardiovascular disease would have met current preventive treatment criteria.

The primary objective of this study was to quantify the population-level treatment gap for LLT using PREVENT-ASCVD risk thresholds. Secondary objectives were to: (1) quantify the burden of fatal CVD occurring without prior diagnosis (primary-prevention fatal CVD), (2) identify predictors of fatal CVD among individuals without baseline disease, and (3) compare phenotypic and treatment profiles between primary-prevention and known fatal CVD decedents. We hypothesized that primary-prevention gap regarding fatal CVD reflects under-recognition and undertreatment of measurable risk rather than absence of risk.

## Methods

### Study population

We analyzed data from NHANES 1999–2018 linked to mortality outcomes through December 31, 2019. NHANES employs a complex, multistage probability design to yield a representative sample of the noninstitutionalized U.S. population. We restricted analyses to participants aged 30–79 years, consistent with primary prevention risk assessment frameworks. The final analyzed sample included 39,277 participants with complete data on cardiovascular risk factors, baseline CVD status, and mortality outcomes (Fig. 1).

**Fig. 1:**
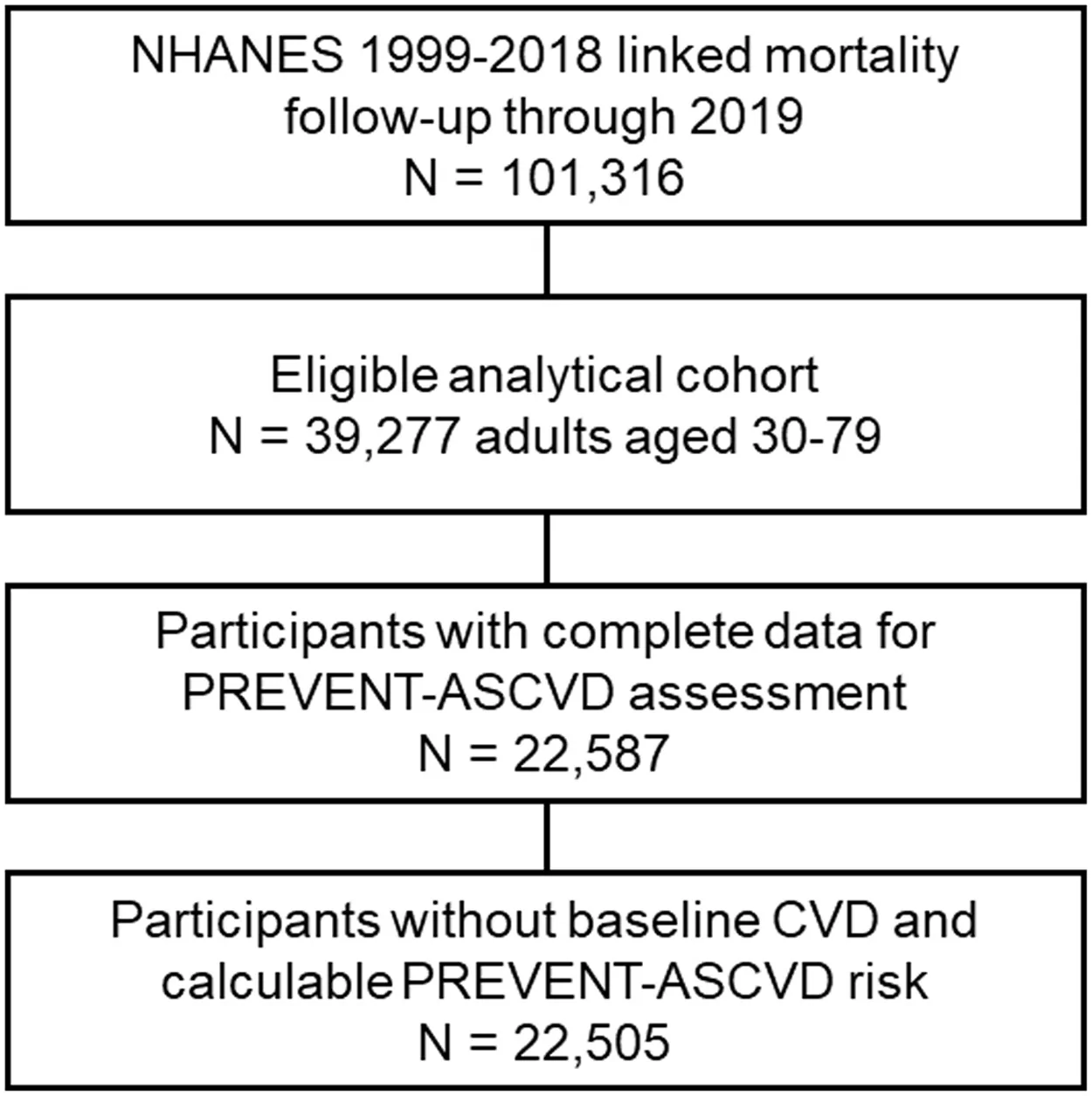
Study Population and Analytical Subset. Legend: Flow diagram illustrating derivation of the analytic cohort from NHANES 1999–2018 participants linked to mortality follow-up through 2019. The figure shows the overall eligible analytic cohort and the subset with complete data for PREVENT-ASCVD risk assessment used in treatment-gap analyses.

As NHANES cycles from 1999 to ​2018 were pooled, examination sample weights were divided by the number of included cycles (n = 10) in accordance with National Center for Health Statistics recommendations. Survey strata and primary sampling units were incorporated throughout all weighted analyses.

### Ethics statement

The NHANES protocols are approved by the National Center for Health Statistics Research Ethics Review Board, and all participants provide written informed consent. The present study used publicly available, de-identified data. An internal review by the U.S. Army Research Institute of Environmental Medicine (USARIEM) Office of Research Quality and Compliance determined that this analysis met criteria for exemption from human subjects research oversight (Protocol #26– 06R)

### Definitions

Diagnosed CVD was defined by self-report of clinician diagnosis of coronary heart disease, myocardial infarction, angina, congestive heart failure, or stroke. Cardiovascular mortality was defined using underlying cause-of-death groupings corresponding to heart disease or cerebrovascular disease. Participants were categorized into four groups: 1) No diagnosed CVD, no CVD death; 2) Diagnosed CVD, no CVD death; 3) No diagnosed CVD, CVD death (primary-prevention fatal CVD); and 4) Diagnosed CVD, CVD death (known fatal CVD).

Lipid-lowering therapy (LLT) was defined as use of statins, ezetimibe, PCSK9 inhibitors, or bempedoic acid. Statins accounted for the overwhelming majority of LLT exposure because PCSK9 inhibitors were not approved until 2015 and bempedoic acid was approved after completion of the study period.

### Covariates

Baseline variables included age, sex, race/ethnicity, education, income-to-poverty ratio, systolic blood pressure, diabetes status, central adiposity, smoking, health insurance, and usual source of care.

### Statistical analysis

Statistical analyses were conducted using R (version 4.4.3; R Foundation for Statistical Computing, Vienna, Austria). All analyses incorporated NHANES complex survey design elements, including sampling weights, strata, and primary sampling units, to generate nationally representative estimates. Burden and incidence rates of fatal cardiovascular disease (CVD) were calculated per 1 000 person-years according to baseline CVD status (Supplementa1 Table S1), and weighted proportions of primary-prevention versus known fatal CVD were estimated. Among participants without baseline CVD, predictors of fatal CVD were evaluated using survey-weighted Cox proportional hazards models to estimate hazard ratios (HRs), with age modeled using restricted cubic splines. PREVENT-ASCVD 10-year risk was calculated using the American Heart Association PREVENT base equations. HbA1c-enhanced and UACR-enhanced equations were not used because these variables were not consistently available across all NHANES cycles. A uniform implementation strategy was used to avoid differential risk characterization across participants. Risk categories were defined according to 2026 multisociety dyslipidemia guidelines as: low (<3%), borderline (3 to <5%), intermediate (5 to <10%), and high (≥10%) risk. The primary treatment-gap analysis used the ≥5% intermediate-or-higher threshold, at which guidelines support a clear indication for LLT initiation; the ≥3% borderline-or-higher threshold was reported as a secondary estimate, reflecting the population for whom LLT is recommended after shared decision-making and consideration of risk-enhancing factors (Blumenthal, 2026). PREVENT-ASCVD thresholds were applied to estimate eligibility for lipid-lowering therapy (LLT), and LLT use was assessed to quantify treatment gaps. Among individuals who experienced fatal CVD, multivariable logistic regression models were used to compare primary-prevention versus known cases. Phenotypic differences between groups were characterized using standardized differences.

To address the evolution of guidelines over the study period, a supplemental analysis was conducted stratifying treatment gaps by era (1999– 2012 vs. 2013– 2018). Additionally, a sensitivity analysis was performed restricting the definition of LLT to statin therapy only, to account for the limited availability of newer non-statin therapies during the study period. Finally, to address potential bias from missing data, we compared the baseline characteristics of participants with and without calculable PREVENT risk.

## Results

Baseline characteristics of the 39,277 U.S. adults included in the descriptive cohort are shown in Table 1. All characteristics reflect baseline measurements obtained at study enrollment. Individuals who experienced primary-prevention fatal CVD were older at baseline than those without CVD events (mean age 62.6 vs 49.1 years), but younger than those with known fatal CVD (67.1 years). Compared with individuals with known fatal CVD, primary-prevention fatal CVD cases demonstrated modestly higher systolic blood pressure and lower prevalence of diabetes. Among adults without baseline diagnosed CVD (n = 34,809), PREVENT-ASCVD risk was calculable in 22,505 participants (64.7%). Characteristics of participants with and without calculable PREVENT risk are shown in Supplemental Table S2. Differences were generally small, with standardized mean differences <0.20 for most characteristics.

**Table 1:** Baseline Characteristics by CVD Status and Outcome.

Characteristic	No CVD / No Death	No CVD / Fatal CVD (Primary Prevention)	CVD / No Death	CVD / Fatal CVD (Known)
Unweighted sample, n	33,846	963	3,825	643
Age, y	49.1 ± 0.1	62.6 ± 0.5	61.1 ± 0.2	67.1 ± 0.5
Body mass index, kg/m²	29.0 ± 0.1	30.0 ± 0.3	31.0 ± 0.2	30.5 ± 0.3
Poverty-income ratio	3.22 ± 0.03	2.54 ± 0.07	2.63 ± 0.04	2.24 ± 0.08
Systolic blood pressure, mmHg	122.8 ± 0.2	136.7 ± 0.9	128.7 ± 0.5	133.2 ± 1.2
Waist circumference, cm	99.1 ± 0.18	104.0 ± 0.75	106.5 ± 0.38	106.0 ± 0.73
Male sex, %	47.3	56.6	55.3	57.7
Diabetes, %	8.2	15.1	23.8	34.5
Current smoking, %	20.7	27.0	25.3	23.5
Insurance coverage, %	83.8	87.4	89.7	96.0
Usual source of care, %	86.3	91.8	95.7	98.1

**Legend:** Baseline characteristics of the study population stratified by baseline cardiovascular disease (CVD) status and fatal CVD outcome. Groups include: (1) no baseline CVD without fatal CVD, (2) no baseline CVD with fatal CVD (primary-prevention fatal CVD), (3) baseline CVD without fatal CVD, and (4) baseline CVD with fatal CVD (known fatal CVD). Continuous variables are presented as weighted means ± standard error, and categorical variables as weighted percentages. All estimates incorporate NHANES survey weights, strata, and primary sampling units. Abbreviations: CVD, cardiovascular disease; PIR, poverty-income ratio; SBP, systolic blood pressure.

### Burden of fatal CVD outside clinical recognition

During follow-up, 1 606 cardiovascular deaths occurred among adults aged 30–79 years. Of these, 38.5% occurred among individuals without baseline diagnosed CVD, whereas 61.5% occurred among those with established baseline disease. Although only 16.5% of the cohort had established baseline CVD, this group experienced substantially higher per-person fatal CVD rates (11.54 vs 0.97 per 1 000 person-years; *P* < 0.001) (Fig. 2).

**Fig. 2:**
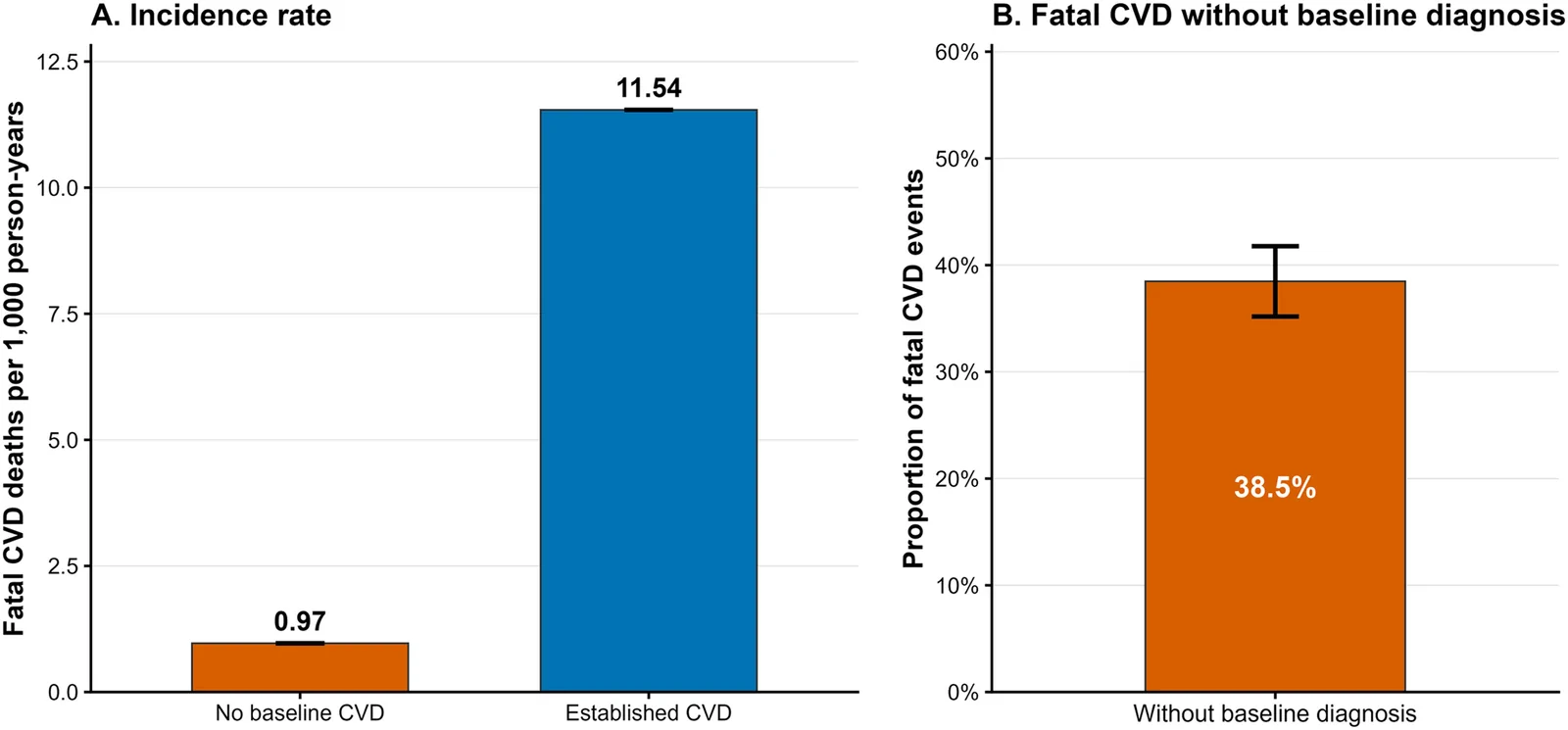
Burden and Incidence of Fatal Cardiovascular Disease by Baseline CVD Status. Legend: (A) Incidence rates of fatal cardiovascular disease according to baseline diagnosed CVD status, presented per 1,000 person-years. (B) Weighted proportion of fatal cardiovascular disease deaths occurring among individuals without baseline diagnosed CVD. Error bars represent 95% confidence intervals.

### Predictors of fatal CVD among adults without baseline diagnosed CVD

Among individuals without baseline CVD, several factors were independently associated with fatal CVD (Table 2; Supplemental Figure S1). Male sex (HR 2.74; 95% CI 1.92–3.92), current smoking (HR 2.55; 95% CI 1.68–3.87), and higher systolic blood pressure (HR 1.01 per mmHg; 95% CI 1.001–1.018) were associated with increased risk. Age demonstrated a strong nonlinear association with mortality risk. In contrast, diabetes was not independently associated after multivariable adjustment.

**Table 2:** Predictors of Primary-Prevention Fatal Cardiovascular Disease (Fully Adjusted Model).

Variable	HR (95% CI)	P value
Male sex	2.74 (1.92–3.92)	<0.001
Poverty-income ratio	0.83 (0.74–0.94)	0.018
Systolic blood pressure (per mmHg)	1.01 (1.001–1.018)	0.050
Waist circumference (per cm)	1.03 (1.017–1.038)	<0.001
Current smoking	2.55 (1.68–3.87)	<0.001
Diabetes	0.75 (0.46–1.22)	0.234
Insurance coverage	0.91 (0.53–1.57)	0.802
Usual source of care	0.85 (0.50–1.44)	0.605

**Legend:** Survey-weighted Cox proportional hazards models estimating hazard ratios (HRs) for fatal cardiovascular disease among individuals without baseline diagnosed CVD. Fully adjusted model including age (modeled using restricted cubic splines), sex, race/ethnicity, education, income-to-poverty ratio, systolic blood pressure, waist circumference, smoking, diabetes, insurance status, and usual source of care. Results are presented as HRs with 95% confidence intervals. Abbreviations: HR, hazard ratio; CI, confidence interval; SBP, systolic blood pressure.

### PREVENT-ASCVD risk and treatment gap

Among individuals meeting PREVENT-ASCVD thresholds, treatment gaps were substantial. Among adults without baseline CVD, 23.6% met the intermediate-or-higher PREVENT-ASCVD risk threshold (≥5%); among these individuals, 67.1% were not receiving LLT. At the broader borderline-or-higher threshold (≥3%), 35.9% met criteria, of whom 71.2% were untreated (Fig. 3; Supplemental Table S3). These findings indicate substantial discordance between estimated cardiovascular risk and treatment exposure. Similar patterns were observed when analyses were stratified according to guideline era (Supplemental Table S4 and Supplemental Figure S2), indicating persistent discordance between retrospectively assigned PREVENT risk and LLT use across both pre-Pooled Cohort Equations (PCE) and post-PCE periods.

**Fig. 3:**
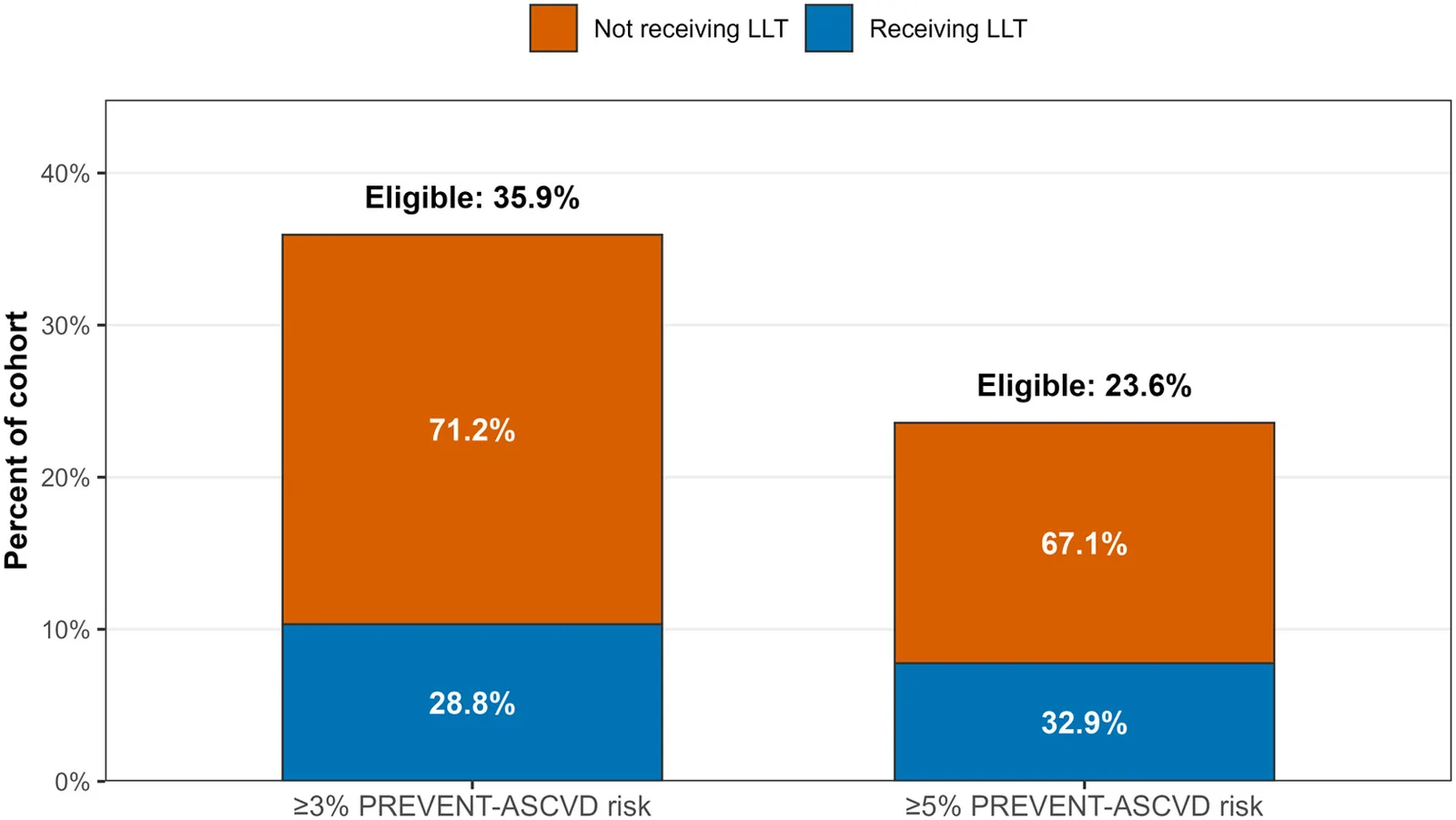
PREVENT-ASCVD eligibility and LDL-lowering therapy treatment gap. Legend: Bars represent the weighted percentage of adults without baseline cardiovascular disease meeting PREVENT-ASCVD risk thresholds of ≥3% and ≥5%. Each bar is divided according to LDL-lowering therapy status among eligible adults. At the ≥5% intermediate-or-higher threshold, 23.6% of adults were eligible for LDL-lowering therapy consideration, of whom 67.1% were not receiving therapy. At the ≥3% borderline-or-higher threshold, 35.9% were eligible, of whom 71.2% were untreated.

### Comparison of primary-prevention vs known fatal CVD

Among individuals who experienced fatal CVD, multivariable analyses demonstrated differences between primary-prevention and known cases (Table 3). Primary-prevention fatal CVD was associated with higher systolic blood pressure and higher socioeconomic status, while diabetes was substantially less prevalent; other traditional risk factors were similar between groups.

**Table 3:** Characteristics of Primary-Prevention vs Known Fatal Cardiovascular Disease.

Variable	OR (95% CI)	P value
Male sex	0.98 (0.52–1.83)	0.946
Poverty-income ratio	1.36 (1.03–1.79)	0.036
Systolic blood pressure (per mmHg)	1.026 (1.006–1.047)	0.014
Waist circumference (per cm)	1.00 (0.987–1.019)	0.755
Diabetes	0.14 (0.07–0.30)	<0.001
Insurance coverage	1.09 (0.29–4.11)	0.897
Usual source of care	0.36 (0.11–1.20)	0.105

**Legend:** Survey-weighted logistic regression models comparing individuals with primary-prevention versus known fatal cardiovascular disease (CVD), restricted to participants who experienced fatal CVD. Odds ratios (ORs) represent the likelihood of primary-prevention versus known status. Fully adjusted model including demographic, clinical, and healthcare access variables. Abbreviations: OR, odds ratio; CI, confidence interval.

### Phenotypic differences between primary-prevention and known fatal CVD

Phenotypic profiling demonstrated broadly similar distributions of cardiometabolic risk factors between primary-prevention and known fatal CVD (Fig. 4). Standardized difference analysis showed modest differences across most traditional risk factors, with larger differences observed for diabetes status, socioeconomic indicators, and treatment-related variables, including lipid-lowering therapy use, insurance coverage, and usual source of care.

**Fig. 4:**
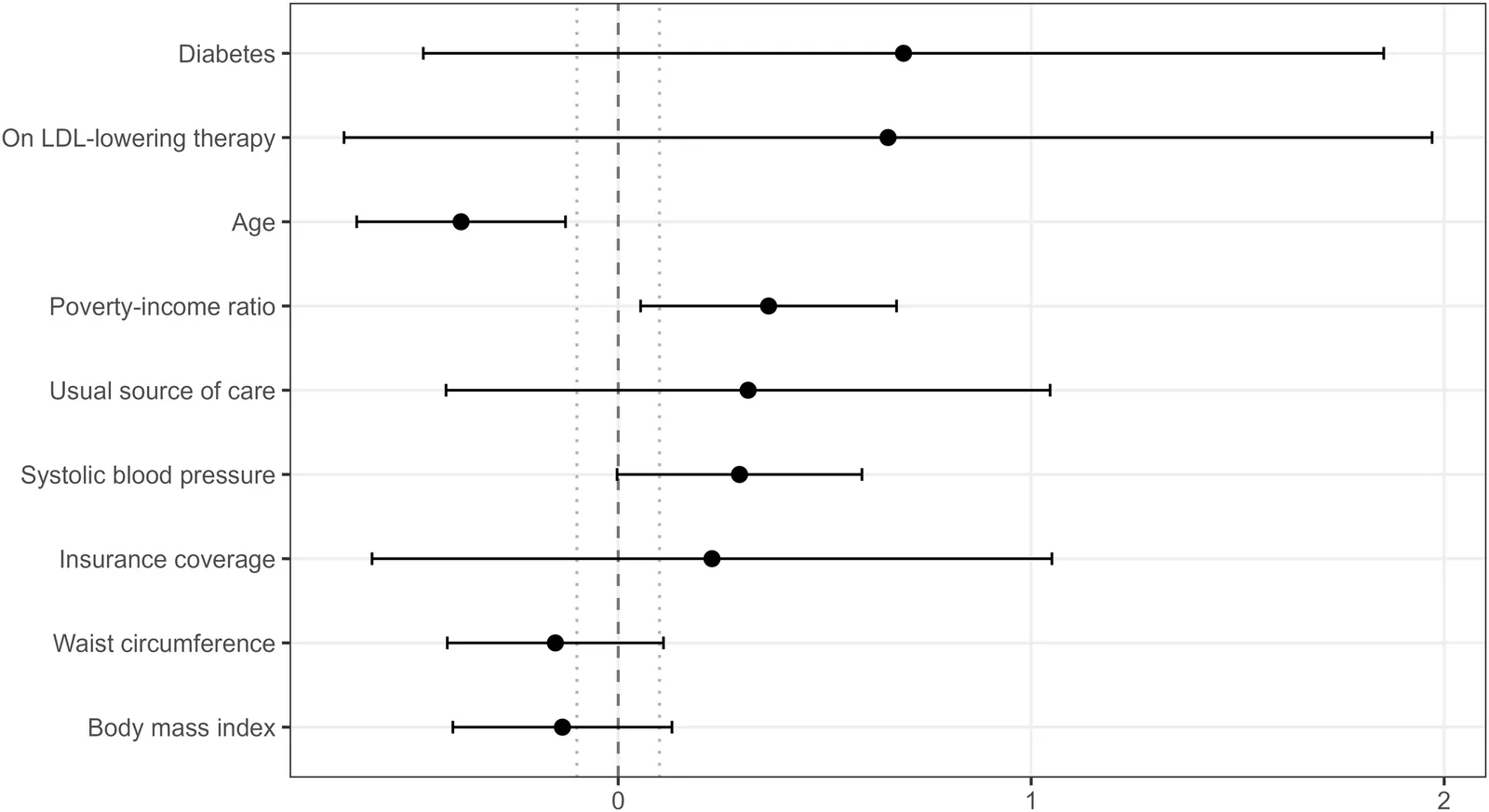
Phenotypic Differences Between Primary-Prevention and Known Fatal Cardiovascular Disease. Legend: Standardized differences in clinical, demographic, and treatment characteristics comparing individuals with primary-prevention versus known fatal cardiovascular disease (CVD). Positive values indicate higher prevalence or higher mean values in primary-prevention fatal CVD, whereas negative values indicate higher values in known CVD. Dashed vertical lines at ±0.10 denote thresholds for meaningful differences. Phenotypic profiles were broadly similar between groups, with modest differences across most traditional cardiometabolic risk factors. Larger differences were observed for diabetes status, socioeconomic indicators, and treatment-related variables, including lipid-lowering therapy use and healthcare access measures, supporting a pattern of under-recognition and undertreatment rather than absence of measurable risk.

### Sensitivity analysis

In sensitivity analyses excluding deaths occurring within the first 2 years of follow-up (Supplemental Table S5), results were materially unchanged. Associations for male sex, systolic blood pressure, current smoking, and socioeconomic status remained consistent in magnitude and direction, supporting the robustness of the primary findings and suggesting that reverse causation from pre-terminal illness did not materially influence the results.

As a sensitivity analysis, Fine-Gray subdistribution hazard models were fit among participants without baseline diagnosed CVD to account for competing noncardiovascular death. Because these models do not incorporate complex survey design, they are presented as supportive analyses. Results were materially unchanged in competing-risk analyses using Fine-Gray models (Supplemental Tables S6 and S7). Male sex, current smoking, higher systolic blood pressure, and greater waist circumference remained associated with fatal CVD. Notably, the association for male sex was attenuated compared with the primary Cox model (sHR 1.69 vs HR 2.74), suggesting that part of the observed association may reflect differential competing mortality rather than cardiovascular mortality alone.

Findings were materially unchanged when treatment exposure was restricted exclusively to statin therapy. Estimates of treatment discordance differed by less than two percentage points across all PREVENT thresholds, indicating that inclusion of nonstatin therapies had minimal influence on study conclusions (Supplemental Table S8).

## Discussion

Among 39,277 U.S. adults followed for mortality, 38.5% (95% CI 35.2–41.8) of fatal cardiovascular disease events occurred among individuals without baseline diagnosed CVD, whereas 61.5% occurred among those with established disease. These findings demonstrate that fatal CVD occurring outside prior clinical recognition represents a major population-level phenomenon rather than an uncommon presentation. Although incidence rates were substantially higher among individuals with established baseline CVD, the large proportion of fatal events arising in adults without diagnosed disease highlights persistent gaps in primary prevention.

Notably, many of these individuals appeared identifiable using contemporary risk stratification frameworks. Among participants without baseline CVD and calculable PREVENT-ASCVD risk (n = 22,505), 35.9% met borderline-or-higher risk criteria (≥3%), including 23.6% meeting the intermediate-or-higher threshold (≥5%) for LDL-lowering therapy consideration. However, 71.2% and 67.1% of these groups, respectively, were not receiving LDL-lowering therapy, representing one of the first nationally representative estimates of the preventive treatment gap under the PREVENT framework. These findings suggest that many individuals who subsequently experienced fatal cardiovascular events had measurable risk factors that would qualify for preventive treatment under contemporary PREVENT-based frameworks.

The principal finding of this study is that retrospective application of contemporary PREVENT-based thresholds identifies a substantial number of individuals who subsequently experienced fatal cardiovascular events and who were not receiving LLT at baseline. Among individuals meeting intermediate or higher risk criteria (≥5% 10-year risk), 23.6% of the population met treatment thresholds, of whom 67.1% were not receiving LLT. Although treatment rates improved at higher levels of risk, a substantial proportion of individuals at clinically actionable risk remained untreated. This gap is not merely theoretical; it is reflected in downstream mortality patterns. A substantial proportion of all fatal CVD events (38.5%; 95% CI 35.2–41.8) occurred in individuals without prior diagnosed cardiovascular disease, the population in whom prevention has its greatest unrealized potential. Although individuals with established CVD exhibited markedly higher absolute risk (11.54 vs 0.97 per 1 000 person-years; *P* < 0.001), the substantially larger undiagnosed population contributed a substantial share of total deaths [2,9]. These findings define a prevention system where the tools exist, the risk is detectable, and the deaths are nonetheless potentially preventable.

Interpretation of these findings requires consideration of the historical context of the study period. Participants were enrolled between 1999 and 2018, spanning multiple generations of cholesterol-management guidelines, including periods preceding both the Pooled Cohort Equations and PREVENT risk assessment frameworks. Consequently, observed treatment discordance should not be interpreted as failure to implement contemporary recommendations, but rather as a retrospective estimate of how current risk assessment strategies would classify individuals observed during earlier prevention eras.

### Primary-prevention fatal CVD as a population-level phenomenon

The observation that a substantial proportion of fatal CVD events occur among individuals without baseline diagnosed disease reinforces longstanding evidence that major cardiovascular events may represent the first clinical manifestation of disease [[3], [10]]. The marked difference in incidence rates between individuals with and without established CVD, coupled with similar total event counts, illustrates the prevention paradox in this population. The 9-fold higher per-person rate of fatal CVD among individuals with diagnosed disease (11.54 vs 0.97 per 1 000 person-years) confirms that established CVD remains the highest-density target for cardiovascular care and should not be deprioritized; rather, the data indicates that the diagnosed and undiagnosed populations require different prevention strategies operating in parallel. Individuals with diagnosed CVD constitute a small, high-risk-density group in whom intensive secondary prevention yields large per-person benefits. Individuals without diagnosed disease constitute a much larger, lower-density group in whom even modest improvements in risk recognition and treatment rates can prevent a substantial absolute number of events because of the size of the population at risk. Strategies focused solely on clinically recognized high-risk populations will therefore fail to capture a large share of potentially preventable cardiovascular mortality, even as such strategies remain essential for those already diagnosed.

Importantly, individuals who experienced primary-prevention fatal CVD were not characterized by absence of risk. Among adults without baseline diagnosed CVD, male sex, current smoking, and higher systolic blood pressure were strong independent predictors of fatal CVD, with a graded, approximately linear association across blood pressure levels. These findings are consistent with large-scale pooled analyses demonstrating a graded association between systolic blood pressure and vascular mortality without a clear threshold [11] and reinforce current guideline recommendations emphasizing early and sustained blood pressure control [12].

Interestingly, diabetes was not independently associated with fatal CVD after multivariable adjustment, and its prevalence was lower among decedents without prior diagnosis compared with those with known CVD. While diabetes is a well-established cardiovascular risk factor [13,14], this finding may reflect differences in clinical recognition and management. Diabetes was not independently associated with fatal CVD in the fully adjusted Cox model, although estimates differed in competing-risk analyses. Specifically, the point estimate shifted from HR 0.75 in the cause-specific model to sHR 1.15 in Fine-Gray analyses. Although neither estimate was statistically significant, this directional change suggests sensitivity to competing mortality and residual confounding. Therefore, these findings should not be interpreted as evidence that diabetes reduces cardiovascular risk.

A key finding of this study is that differences between primary-prevention and known fatal CVD are driven primarily by patterns of diagnosis and treatment rather than by substantial differences in underlying risk factor burden. Phenotypic profiling demonstrated broadly similar distributions of major risk factors between groups, with the most pronounced differences reflecting treatment exposure and clinical labeling. Consistent with this interpretation, healthcare access variables, including insurance status and usual source of care, were not independently associated with primary-prevention status after adjustment. Although prior studies have linked healthcare access to cardiovascular outcomes [15,16], our findings suggest that the critical gap lies not in access alone, but in the recognition and management of existing risk within the healthcare system. Collectively, suggest that many individuals experiencing fatal CVD without prior diagnosis had measurable cardiometabolic risk factors before death.

These findings reframe where the gap in cardiovascular prevention actually lies. The PREVENT-ASCVD calculator, now the standard for primary prevention risk assessment, identified a substantial portion of the adult population as meeting threshold for considering LLT, yet 71% of those individuals were untreated. These findings highlight a gap between risk identification and treatment implementation. As PREVENT thresholds and corresponding treatment recommendations are newly established, some of this gap likely reflects recent changes in guideline definitions. However, the magnitude of undertreatment observed suggests that translation of risk into therapy remains incomplete at the population level. The downstream consequence is measurable in this dataset: 38.5% of cardiovascular deaths occurring outside clinical diagnosis. These findings raise the possibility that improved translation of cardiovascular risk assessment into preventive treatment could reduce future cardiovascular disease burden, although prospective implementation studies are needed to establish causality. Blood pressure control, smoking cessation, and reduction of central adiposity remain the dominant modifiable drivers of fatal events in this group, and all are actionable without awaiting a formal CVD diagnosis. Current guidelines support this approach [12]; the implementation gap documented here suggests that guideline adherence, not guideline content, is the binding constraint.

Several limitations warrant consideration. Baseline CVD status was based on self-reported clinician diagnosis, without medication-adjudicated or claims-based confirmation. A single-item self-report is likely to misclassify a non-trivial share of individuals with prior MI, revascularization, or heart failure as having no diagnosed CVD, and this misclassification is unlikely to be random: lower socioeconomic status and reduced healthcare engagement are associated with both underdiagnosis and adverse outcomes. The estimate that 38.5% of fatal CVD occurred in individuals without prior diagnosis should therefore be interpreted as a plausible upper bound on true primary-prevention deaths, and the phenotypic comparison between primary-prevention and known fatal CVD groups is likely attenuated by misclassification. Cause-of-death classification from the National Death Index may also introduce error [17]. Residual confounding is possible despite multivariable adjustment, and risk factor assessment was based on single baseline measurements, which may not reflect longitudinal exposure. Comparisons restricted to fatal CVD cases (Table 3) are subject to index-event bias, in which conditioning on the outcome can induce associations between exposures and disease-recognition status; the markedly lower odds of diabetes among primary-prevention decedents likely reflects this mechanism rather than a true protective effect, since diabetes screening reliably triggers cardiovascular workup and reduces the likelihood that progressive CVD remains undiagnosed. Although competing risks from noncardiovascular mortality were evaluated in sensitivity analyses using Fine-Gray models, these analyses were conducted without incorporation of complex survey weighting and should be interpreted as supportive rather than primary findings. Lastly, as PREVENT thresholds were retrospectively applied to historical cohorts, treatment eligibility estimates should be interpreted as counterfactual assessments rather than evaluations of guideline adherence during the original observation period. Despite these limitations, the use of nationally representative data with standardized survey weighting and long-term mortality follow-up enhances generalizability and provides robust contemporary estimates.

In this nationally representative analysis, nearly 40% of fatal cardiovascular events occurred in individuals without baseline diagnosed CVD. Among adults meeting the primary PREVENT-ASCVD intermediate-or-higher risk threshold, approximately two-thirds were not receiving lipid-lowering therapy. These findings suggest that many individuals who subsequently experienced fatal cardiovascular disease would have met contemporary PREVENT-based treatment thresholds when assessed retrospectively. Future prospective studies are needed to determine whether systematic implementation of PREVENT-guided preventive therapies can reduce cardiovascular mortality.

## Declaration of generative AI use

During the preparation of this work the authors used artificial intelligence tools (OpenAI ChatGPT image-generation capabilities) to assist in the iterative development and refinement of the central illustration. After using this tool, the authors reviewed and edited the content as needed and take full responsibility for the content of the published article.

The authors have no conflicts of interest.

## Funding

Research was funded by the Military Operational Medicine Research Program, Fort Detrick, MD. The funding source had no role in study design; collection, analysis, and interpretation of data; writing of the report; and has no restrictions regarding the submission of the report for publication.

## Author disclosures

Disclaimer: The opinions or assertions contained herein are the private views of the author(s) and are not to be construed as official or reflecting the views of the Army or Department of War. Any citations of commercial organizations and trade names in this report do not constitute an official Department of the Army endorsement of approval of the products or services of these organizations. The investigators have adhered to the policies for protection of human participants as prescribed in DODI 3216.02, and the research was conducted in adherence with the provisions of 32 CFR Part 219.

## Ethics statement

The NHANES protocols are approved by the National Center for Health Statistics Research Ethics Review Board, and all participants provide written informed consent. The present study used publicly available, de-identified data. An internal review by the U.S. Army Research Institute of Environmental Medicine (USARIEM) Office of Research Quality and Compliance determined that this analysis met criteria for exemption from human subjects research oversight (Protocol #26– 06R)

## CRediT authorship contribution statement

**Adam W. Potter:** Writing – review & editing, Writing – original draft, Formal analysis, Data curation, Conceptualization. **Caroline E. Murphy:** Writing – review & editing, Writing – original draft, Methodology, Formal analysis, Conceptualization. **Bradley J. Petek:** Writing – review & editing, Writing – original draft, Methodology, Conceptualization. **Christopher L. Chapman:** Writing – review & editing, Writing – original draft, Formal analysis, Conceptualization. **Karl E. Friedl:** Writing – review & editing, Writing – original draft, Supervision. **Timothy W. Churchill:** Writing – review & editing, Supervision, Methodology. **Nisha Charkoudian:** Writing – review & editing, Writing – original draft, Supervision, Conceptualization.

## Declaration of competing interest

The authors have no conflicts of interest.

Associate Editor role: BJP is currently an Associate Editor for AJPC. He acknowledges he will have no involvement in the peer-review process for this article and that responsibility for evaluating this manuscript and selecting reviewers has been formally delegated to another editor

## References

[1] Di Cesare M., Bixby H., Gaziano T., Hadeed L., Kabudula C., McGhie D.V. (2023).

[2] Martin S.S., Aday A.W., Allen N.B., Almarzooq Z.I., Anderson C.A., Arora P. (2025). 2025 heart disease and stroke statistics: a report of US and global data from the American Heart Association. Circulation.

[3] Myerburg R.J., Junttila M.J. (2012). Sudden cardiac death caused by coronary heart disease. Circulation.

[4] Sara J.D., Eleid M.F., Gulati R., Jr H.D.R. (2014). Sudden cardiac death from the perspective of coronary artery disease. Mayo Clin Proc.

[5] Rose G. (2001). Sick individuals and sick populations. Int J Epidemiol.

[6] Khan S.S., Matsushita K., Sang Y., Ballew S.H., Grams M.E., Surapaneni A. (2024). Development and validation of the American Heart Association’s PREVENT equations. Circulation.

[7] Blumenthal R.S., Morris P.B., Gaudino M., Akl E.A., Bittencourt M.S., Borden W.B. (2026). 2026 ACC/AHA/AACVPR/ABC/ACPM/ADA/AGS/APhA/ASPC/NLA/PCNA guideline on the management of dyslipidemia: a report of the American College of Cardiology/American Heart Association Joint Committee on Clinical Practice Guidelines. J Am Coll Cardiol.

[8] Dattani S., Samborska V., Ritchie H., Roser M. (2026). Cardiovascular diseases. Our world in data. https://ourworldindata.org/cardiovascular-diseases.

[9] Tsao C.W., Aday A.W., Almarzooq Z.I., Anderson C.A., Arora P., Avery C.L. (2023). Heart disease and stroke statistics—2023 update: a report from the American Heart Association. Circulation.

[10] Kannel W.B., Plehn J.F., Cupples L.A. (1988). Cardiac failure and sudden death in the Framingham Study. Am Heart J.

[11] Prospective Studies Collaboration (2007). Blood cholesterol and vascular mortality by age, sex, and blood pressure: a meta-analysis of individual data from 61 prospective studies with 55 000 vascular deaths. Lancet.

[12] Arnett D.K., Blumenthal R.S., Albert M.A., Buroker A.B., Goldberger Z.D., Hahn E.J. (2019). 2019 ACC/AHA guideline on the primary prevention of cardiovascular disease: a report of the American College of Cardiology/American Heart Association Task Force on Clinical Practice Guidelines. J Am Coll Cardiol.

[13] Emerging Risk Factors Collaboration (2011). Diabetes mellitus, fasting glucose, and risk of cause-specific death. N Engl J Med.

[14] Shah A.D., Langenberg C., Rapsomaniki E., Denaxas S., Pujades-Rodriguez M., Gale C.P. (2015). Type 2 diabetes and incidence of cardiovascular diseases: a cohort study in 1.9 million people. Lancet Diabetes Endocrinol.

[15] Baker D.W., Sudano J.J., Albert J.M., Borawski E.A., Dor A. (2001). Lack of health insurance and decline in overall health in late middle age. N Engl J Med.

[16] Wilper A.P., Woolhandler S., Lasser K.E., McCormick D., Bor D.H. (2009). Himmelstein DU. Health insurance and mortality in US adults. Am J Public Health.

[17] Chandramohan D., Setel P., Quigley M. (2001). Effect of misclassification of causes of death in verbal autopsy: can it be adjusted?. Int J Epidemiol.

