# Supplementary material for: Risk–Treatment Discordance and Fatal Cardiovascular Disease: A Nationwide Analysis Using PREVENT Thresholds
Source: Am J Prev Cardiol. 2026 Jun 20;30:101711. doi: 10.1016/j.ajpc.2026.101711 (PMC13632123; doi:10.1016/j.ajpc.2026.101711)
Supplement: Supplementary file 1 [file mmc1.docx]

**Supplemental Materials**

**Supplemental Table S1. Burden and Incidence of Fatal Cardiovascular Disease by Baseline CVD Status**

| Metric | | Estimate (95% CI) | |
| --- | --- | --- | --- |
| Proportion of fatal CVD without prior diagnosis | | 38.5 (35.2-41.8) | |
|  | | | |
| Baseline CVD Status | **Deaths (weighted)** | **Person-Years** | **Incidence rate (per 1,000 PY)** |
| No baseline CVD | 1,389,991 | 1,428,475,697 | 0.97 |
| Established CVD | 1,165,182 | 100,749,387 | 11.54 |

**Legend:** Weighted proportion and incidence rates of fatal cardiovascular disease (CVD) according to baseline diagnosed CVD status. Incidence rates are expressed per 1,000 person-years. Primary-prevention fatal CVD is defined as fatal CVD occurring in individuals without baseline diagnosed disease. All estimates account for NHANES complex survey design.

**Supplemental Table S2. Characteristics of Participants With and Without Calculable PREVENT-ASCVD Risk.**

| Characteristic | PREVENT Calculable | PREVENT Not Calculable | Standardized Difference |
| --- | --- | --- | --- |
| Age, years | 49.8 | 48.5 | 0.11 |
| BMI, kg/m² | 29.3 | 28.5 | 0.12 |
| Poverty-income ratio | 3.2 | 3.1 | 0.08 |
| Systolic blood pressure, mmHg | 122.6 | 124.1 | -0.09 |
| Waist circumference, cm | 100.1 | 97.5 | 0.16 |
| Total cholesterol, mg/dL | 200.4 | 197.7 | 0.07 |
| HDL cholesterol, mg/dL | 54.1 | 53.8 | 0.02 |
| Male sex, % | 52.3 | 53.0 | -0.01 |
| Diabetes, % | 8.0 | 12.0 | 0.13 |
| Current smoking, % | 19.1 | 24.3 | 0.13 |
| Insurance coverage, % | 84.0 | 82.9 | -0.03 |
| Usual source of care, % | 86.3 | 86.7 | 0.01 |
| Lipid-lowering therapy, % | 15.4 | 6.1 | -0.31 |
| Fatal CVD during follow-up, % | 0.9 | 4.2 | 0.21 |
| All-cause death during follow-up, % | 3.8 | 17.5 | 0.46 |

**Legend:** Among 34,809 adults without baseline cardiovascular disease, PREVENT-ASCVD risk could be calculated for 22,505 participants (64.7%) and could not be calculated for 12,304 participants (35.3%).

**Supplemental Table S3. Preventive Treatment Eligibility and LLT Treatment Gap**

| Metric | Estimate |
| --- | --- |
| Borderline or Higher PREVENT-ASCVD risk (≥3% risk) | 35.9% (n=8,089) |
| Intermediate or Higher PREVENT-ASCVD risk (≥5% risk) | 23.6% (n=5,306) |
| Untreated with LLT among Borderline or Higher PREVENT-ASCVD risk (≥3%) | 71.2% (n=5,763) |
| Untreated with LLT among Intermediate or Higher PREVENT-ASCVD risk (≥5%) | 67.1% (n=3,558) |

| PREVENT Risk Category | Unweighted N | Weighted Population | Cohort % | Treated (%) | Untreated (%) |
| --- | --- | --- | --- | --- | --- |
| Low (<3%) | 11,246 | 54.4 million | 64.0 | 7.1 | 92.9 |
| Borderline (3 to <5%) | 2,783 | 12.8 million | 15.1 | 22.4 | 77.6 |
| Intermediate (5 to <10%) | 3,465 | 12.4 million | 14.7 | 31.6 | 68.4 |
| High (≥10%) | 1,841 | 5.3 million | 6.2 | 36.4 | 63.6 |

**Legend:** Proportion of participants meeting PREVENT-ASCVD risk thresholds and corresponding LLT use. Estimates reflect weighted population proportions. Treatment gap defined as proportion of eligible individuals not receiving LLT therapy. Abbreviations: CVD, cardiovascular disease. Using PREVENT risk thresholds, 6.2% of U.S. adults without baseline cardiovascular disease were classified as high risk (≥10%), representing approximately 5.3 million individuals nationally. Despite qualifying for preventive therapy, 63.6% of high-risk adults were not receiving lipid-lowering treatment.

**Supplemental Table S4. Treatment Gap by Guideline Era**

| Era | Threshold | Eligible (%) | Treated (%) | Untreated (%) |
| --- | --- | --- | --- | --- |
| 1999–2012 | ≥3% | 33.3 | 74.2 | 25.8 |
| 2013–2018 | ≥3% | 39.2 | 68.6 | 31.4 |
| 1999–2012 | ≥5% | 18.8 | 69.4 | 30.6 |
| 2013–2018 | ≥5% | 23.5 | 64.5 | 35.5 |
| 1999–2012 | ≥10% | 5.6 | 64.3 | 35.7 |
| 2013–2018 | ≥10% | 7.0 | 63.0 | 37.0 |

**Legend:** Treatment discordance remained evident across both guideline eras. Approximately one-third of adults meeting contemporary PREVENT treatment thresholds remained untreated during both the pre-PCE (1999–2012) and post-PCE (2013–2018) periods, suggesting persistent implementation gaps despite evolving cardiovascular risk assessment frameworks.

**Supplemental Table S5. Sensitivity Analysis Excluding Early Deaths (First 2 Years)**

| **Variable** | **HR (95% CI)** | **P value** |
| --- | --- | --- |
| **Male sex** | 2.60 (1.75–3.87) | <0.001 |
| **Poverty-income ratio** | 0.83 (0.73–0.95) | 0.021 |
| **Systolic blood pressure (per mmHg)** | 1.01 (0.998–1.017) | 0.165 |
| **Waist circumference (per cm)** | 1.03 (1.020–1.044) | <0.001 |
| **Current smoking** | 2.65 (1.64–4.26) | <0.001 |
| **Diabetes** | 0.92 (0.56–1.53) | 0.761 |
| **Insurance coverage** | 0.93 (0.51–1.72) | 0.869 |
| **Usual source of care** | 0.62 (0.36–1.08) | 0.170 |

**Legend:** Survey-weighted Cox proportional hazards models estimating hazard ratios (HRs) for fatal cardiovascular disease among individuals without baseline diagnosed CVD, excluding deaths occurring within the first 2 years of follow-up. Models adjusted for age (restricted cubic splines), sex, race/ethnicity, education, income-to-poverty ratio, systolic blood pressure, waist circumference, smoking, diabetes, insurance status, and usual source of care. Results were materially unchanged compared with the primary analysis.

**Supplemental Table S6. Competing-risk sensitivity analysis using Fine-Gray models among participants without baseline diagnosed CVD**

| **Variable** | **sHR (95% CI)** | **P value** |
| --- | --- | --- |
| **Male sex** | 1.69 (1.27–2.25) | <0.001 |
| **Poverty-income ratio** | 0.78 (0.70–0.88) | <0.001 |
| **Systolic blood pressure (per mmHg)** | 1.01 (1.01–1.02) | <0.001 |
| **Waist circumference (per cm)** | 1.02 (1.01–1.03) | <0.001 |
| **Current smoking** | 2.30 (1.67–3.17) | <0.001 |
| **Diabetes** | 1.15 (0.81–1.64) | 0.436 |
| **Insurance coverage** | 1.04 (0.64–1.71) | 0.864 |
| **Usual source of care** | 1.09 (0.65–1.82) | 0.751 |

**Legend:** Subdistribution hazard ratios (sHRs) were estimated using Fine-Gray competing-risk models among adults without baseline diagnosed cardiovascular disease, with fatal cardiovascular disease as the event of interest and noncardiovascular death treated as the competing event. Models included the same covariates as the primary fully adjusted Cox model: age (modeled using restricted cubic splines), sex, race/ethnicity, education, income-to-poverty ratio, systolic blood pressure, waist circumference, smoking, diabetes, insurance status, and usual source of care. Results were directionally and materially consistent with the primary survey-weighted Cox models.

**Supplemental Table S7. Cause-Specific Cox and Fine-Gray Competing Risk Models**

| Variable | Cox HR (95% CI) | p | Fine-Gray sHR (95% CI) | p |
| --- | --- | --- | --- | --- |
| Male sex | 2.74 (1.92–3.92) | <0.001 | 1.69 (1.27–2.25) | <0.001 |
| Poverty-income ratio | 0.83 (0.74–0.94) | 0.018 | 0.78 (0.70–0.88) | <0.001 |
| Systolic blood pressure | 1.01 (1.00–1.02) | 0.050 | 1.01 (1.01–1.02) | <0.001 |
| Diabetes | 0.75 (0.46–1.22) | 0.234 | 1.15 (0.81–1.64) | 0.436 |
| Waist circumference | 1.03 (1.02–1.04) | <0.001 | 1.02 (1.01–1.03) | <0.001 |
| Current smoking | 2.55 (1.68–3.87) | <0.001 | 2.30 (1.67–3.17) | <0.001 |

**Supplemental Table S8. Sensitivity Analysis Restricting Treatment Exposure to Statin Therapy**

| PREVENT Threshold | LLT Treated (%) | LLT Untreated (%) | Statin Treated (%) | Statin Untreated (%) |
| --- | --- | --- | --- | --- |
| ≥3% | 71.4 | 28.6 | 72.1 | 27.9 |
| ≥5% | 67.0 | 33.0 | 67.9 | 32.1 |
| ≥10% | 63.6 | 36.4 | 65.2 | 34.8 |

**Legend:** Findings were materially unchanged when treatment exposure was restricted exclusively to statin therapy. Estimates of treatment discordance differed by less than two percentage points across all PREVENT thresholds, indicating that inclusion of nonstatin therapies had minimal influence on study conclusions.

**Supplemental Figure S1. Association Between Systolic Blood Pressure and Risk of Fatal Cardiovascular Disease**

**Legend:** Adjusted hazard ratios for fatal cardiovascular disease across the range of systolic blood pressure among individuals without baseline CVD, modeled as a continuous linear term in a survey-weighted Cox proportional hazards model. Hazard ratios are referenced to 120 mmHg. Shaded areas represent 95% confidence intervals. The curve is displayed over the central distribution of systolic blood pressure to avoid unstable estimates in sparsely populated ranges.

**Supplemental Figure S2. Temporal Trends in Lipid-Lowering Therapy Treatment Gaps Across NHANES Cycles**

**Legend:** Percent untreated among adults meeting retrospective PREVENT treatment thresholds across NHANES cycles from 1999–2000 through 2017–2018. Treatment discordance persisted throughout the study period despite evolving cardiovascular prevention guidelines.
